# The association between non-alcoholic fatty liver disease and urinary incontinence among adult females in the United States

**DOI:** 10.1186/s12889-024-18578-8

**Published:** 2024-05-22

**Authors:** Xinyuan Li, Weiwei Zhou, Guangsheng Hu

**Affiliations:** Department of Gastroenterology, The First Affiliated Hospital of University of South China, Hengyang, Hunan 421001 People’s Republic of China

**Keywords:** Urinary incontinence, NAFLD, NHANES, Cross-sectional, United States

## Abstract

**Background and objectives:**

Non-alcoholic fatty liver disease (NAFLD) and urinary incontinence (UI) are both highly prevalent and age-related diseases. Nevertheless, the link between NAFLD and UI is unclear. Hence, the study was designed to evaluate the association between the NAFLD and UI (including UI types) in a nationally representative sample of United States (US) female adults.

**Methods:**

We conducted this study used data from U.S. female adults in the National Health and Nutrition Examination Survey (NHANES) 2017-March 2020 (pre-pandemic) cycles. The diagnosis of NAFLD is based on Vibration controlled transient elastography (VCTE) and absence of know liver diseases and significant alcohol consumption. The diagnosis and types of UI were assessment using a self-report questionnaire. Multivariable logistic regression models were used to analyze the association between NALFD and UI. Stratified analyses based on age, obesity, race, educational level, married status, PIR, and smoking status were conducted.

**Results:**

Of the 2149 participants, the mean (95% CI) age was 53.9 (52.7–55.0), 686 (61.1%) were Non-Hispanic White. UI was significantly more common in participants with NAFLD [490 (64.7%)] than those without NAFLD [552 (44.9%)]. Adjusted for age, race/ethnicity, marital status, educational level, family poverty income ratio (PIR) status, alanine aminotransferase (ALT), aspartate aminotransferase (AST), smoking status, obesity, type 2 diabetes mellitus (T2DM), hypertension and insulin resistance (IR) in a multivariable logistic regression model, NALFD were associated with UI [OR: 1.93, 95%CI 1.23–3.02, *P* = 0.01] and urge UI [OR: 1.55, 95%CI 1.03–2.33, *P* = 0.03], while patients with NAFLD did not show an increased odds in stress UI and mixed UI when compared with those without NAFLD subject (*P* > 0.05). In the subgroup analyses, NAFLD remained significantly associated with UI, particularly among those participants without obesity (OR: 2.69, 95% CI 1.84-4.00) and aged ≥ 60 years (OR: 2.20, 95% CI 1.38–3.51).

**Conclusions:**

Among US female adults, NAFLD has a strong positive correlation with UI. Given that NAFLD is a modifiable disease, these results may help clinicians to target female patients with NAFLD for treatments and interventions that may help prevent the occurrence of UI and reduce the symptoms of UI.

## Introduction

Non-alcoholic fatty liver disease (NAFLD) is a clinico-pathologic syndrome characterized by diffuse bulla fat of hepatocytes (the presence of more than 5% of hepatic steatosis), with the exception of considerable alcohol consumption and other well-defined liver damage factors such as drugs, autoimmune, viral hepatitis, and other causes [1]. NAFLD is the most common chronic liver disease, affecting an estimated 32.4% of adult population and approximately 25.9% of adolescents worldwide [2, 3]. At the same time, its global prevalence is increasing at an alarming rate and is in line with the growing global trend of obesity and type 2 diabetes mellitus (T2DM) [2]. Fueled by the increasing diagnosis of NAFLD, the direct medical costs caused by NAFLD in the United States (US) have exceeded $100 billion annually [4]. As to the treatment of NAFLD, in addition to drug treatment, intensive lifestyle intervention has been proven to be effective in the remission of NAFLD [5, 6].

Urinary incontinence (UI), a common chronic condition that causes psychological distress and worsening quality of life [7]. UI can be divided into three types: stress UI (SUI), urge UI (UUI) and mixed UI (MUI) [8]. The prevalence of UI has increased over the past few decades, affecting approximately 19.3% US male and 45.9%% US female [9, 10]. The main risk factors for UI are include modifiable risks (such as obesity, metabolic syndrome (MetS), T2DM, hypertension, smoking status, the level of physical exercise, etc.) and unmodifiable risk factors (such as advanced age, neurological disease, history of surgery, etc.) [7, 11–13]. Identifying and intervening the modifiable risk factors of UI is an economical and effective scheme to prevent UI, reduce symptoms of UI and reduce adverse events related to UI.

Considering that NAFLD is closely related with obesity and MetS, we hypothesize that the existence of NAFLD may be related to urinary incontinence [14, 15].

Therefore, our objective was to assess the association between NAFLD and UI in a large, nationally representative sample of adult female in the US.

## Methods

### Date sources

NHANES is a cross-sectional research program conducted by the Centers for Disease Control (CDC) and National Center for Health Statistics (NCHS) to assess the health and nutrition status of adults and children in the US. The NHANES program began in the early 1960s and was conducted as a series of surveys focusing on different population groups or health topics. A nationally representative sample of around five thousand people is surveyed annually, and because it oversamples specific age and racial groups, NHANES provides comprehensive data that is representative of the civilian and noninstitutionalized population in the US. The participants provided their written informed consent to participate in this study and their personal information was de-identified in the NHANES database.

### Study design and population

**Fig. 1:**
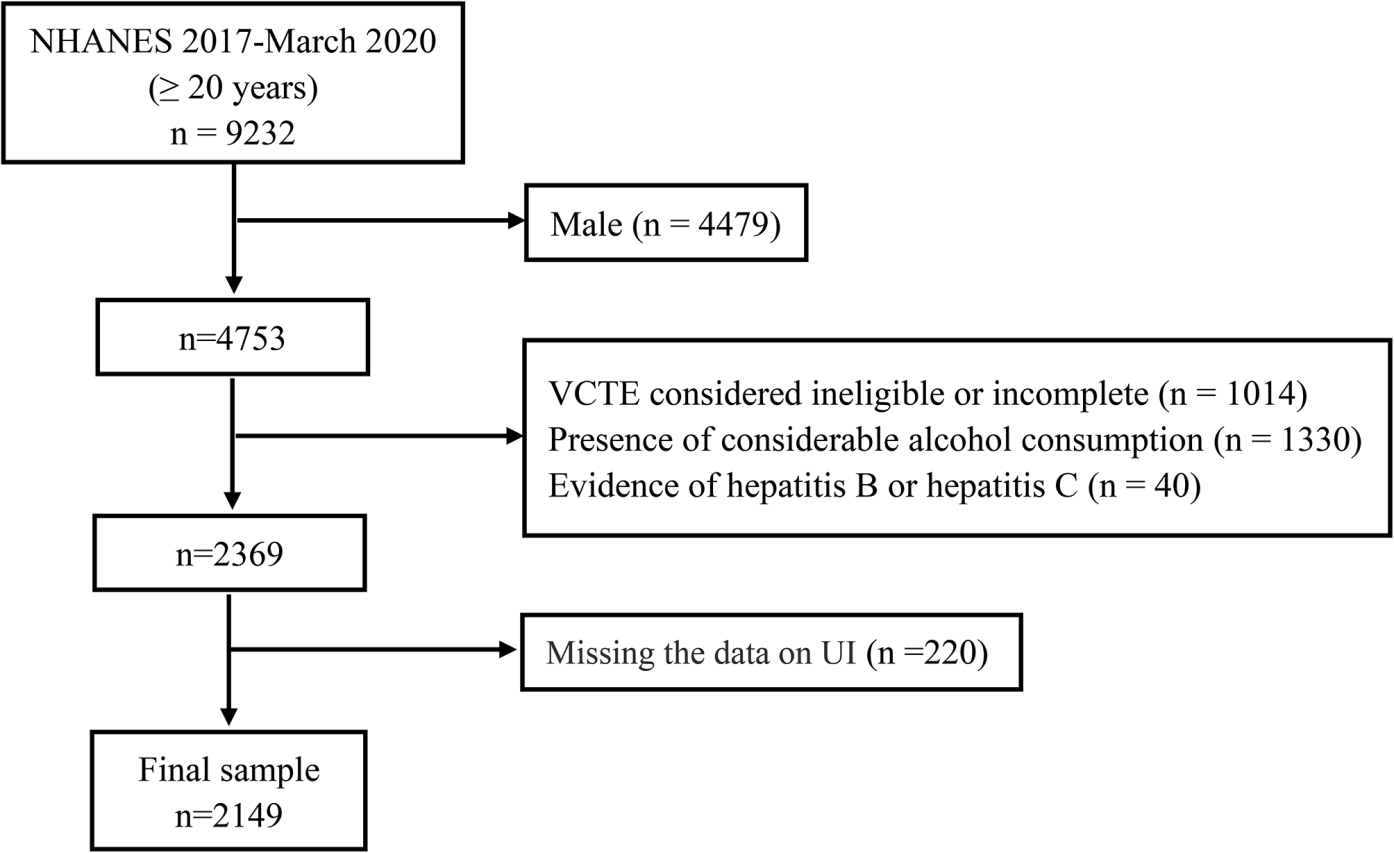
Flowchart showing the selection of eligible participants

Data collected from participants in the 2017-March 2020 (pre-pandemic) NHANES cycle are used in our analysis. The data included demographic data, examination data, laboratory data, and questionnaire data for the presented analysis. In NHANES 2017-March 2020 (pre-pandemic) cycle, the overall sample included 9232 people over 20 years of age. We excluded those individuals with one of the following conditions: (I) males (*n* = 4479); (II) missing or incomplete VCTE test data (*n* = 1014); (III) evidence of considerable alcohol consumption (more than 14 drinks per week (*n* = 1330); (IV) evidence of viral hepatitis (serum hepatitis B surface antigen positive or serum hepatitis C antibody positive) (*n* = 40); (V) missing the data of UI (*n* = 220). In the end, our study included 2149 participants. The screening process flowchart was shown in Fig. 1.

### Diagnosis of NAFLD

Despite the fact that liver tissue biopsy evaluation is recommended by clinical guidelines as the golden standard for diagnosing hepatic disease, it is impractical to perform liver biopsy examination to evaluate liver disease status (such as hepatic steatosis) for large populations, given the current global prevalence of patients with NAFLD [1, 16, 17]. Vibration controlled transient elastography (VCTE) is a widely used non-invasive and convenient method to identify hepatic steatosis through the value of controlled attenuation parameter (CAP) [18, 19]. In the NHANES 2017-March 2020 (pre-pandemic) cycle, the VCTE was carried out in the Mobile Examination Center using an ultrasound machine (FibroScan®502 V2 Touch instrument). The elastography exam was performed by NHANES health technicians (HTs), who were trained and certified by NHANES staff, Westat and the equipment manufacturer (Echosens™ North America). In the presented study, individuals were diagnosed with hepatic steatosis by CAP ≥ 274 dB/m, as this threshold highly showed accuracy in identifying hepatic steatosis [20, 21]. If the average alcoholic drink per week is more than 14 drinks per week, it was considered considerable alcohol consumption [1]. Viral hepatitis was defined as either viral hepatitis B (positive for serum hepatitis B surface antigen test) or hepatitis C (positive for serum hepatitis C antibody test).

### Diagnosis of UI

The information on urinary incontinence in NHANES was collected from kidney conditions only for people over 20 years of age. If participants answered “never” to this question “How often have urinary leakage?”, they were defined as UI [22].

Answering “yes” to the following question was defined as the SUI: “During the past 12 months, have you leaked or lost control of even a small amount of urine with activity like coughing, lifting, or exercise?”. Answering “yes” to the following question was defined as the UUI: “During the past 12 months, have you leaked or lost control of even a small amount of urine with an urge or pressure to urinate and you couldn’t get to the toilet fast enough?”. Those who were diagnosed with both SUI and UUI were defined as MUI.

### Covariates

On basis of the literature, the potential confounders included age, ethnicity, marital status, educational level, family poverty income ratio (PIR) status, alanine aminotransferase (ALT), aspartate aminotransferase (AST), smoking status (never, current, or former), obesity (yes or no), T2DM (yes or no), hypertension (yes or no), insulin resistance (IR) (yes or no). The smoking status was categorized as current smoker (had smoked ≥ 100 cigarettes in their lifetime and smoking everyday/somedays), former smoker (had smoked ≥ 100 cigarettes in their lifetime but not smoking now), and never smoker (had smoked < 100 cigarettes in their lifetime). The PIR status divided into 3 levels: low income (≤ 1.3), medium income (≥ 1.3 to 3.5), and high income (> 3.5) [23]. The body mass index (BMI) cut-off 30 kg/m^2^ was used to define obesity. According to the American Diabetes Association criteria, participants were diagnosed with (T2DM) if they met any of the following conditions: (I) a self-reported history of diagnosis; (II) use of antidiabetic drugs (consists of oral antidiabetic drugs or insulin); (III) glycated hemoglobin A1c (HbA1c) level ≥ 6.5%; (IV) fasting plasma glucose ≥ 7.0 mmol/L [24]. Hypertension was diagnosed when participants had a systolic blood pressure (SBP) ≥ 140 mmHg and/or diastolic blood pressure (DBP) ≥ 90 mmHg or had a self-reported history of hypertension, or self-reported current use of antihypertensive drugs [25]. IR was assessed by using HOMA-IR model, which was calculated as [(fasting insulin (µU/mL) × fasting glucose (mmol/L) ]/22.5 [26].

### Statistical analysis

All statistical analyses were performed using R software (version 4.2.0). *P* value < 0.05 was considered statistically significant. We selected appropriate weights for each analysis in this study, as recommended by the NCHS. Baseline characteristics were described as mean (95% confidence intervals) for continuous variables and numbers (percentages) for categorical variables. Student-t tests was used to compare continuous data and χ2 test was used to compare categorical data. Logistic regression models were used to estimate the odd ratios (ORs) and 95% confidence intervals (CIs) for the association of NAFLD with UI. In the multivariable models, age (continuous), race/ethnicity (Mexican American, other Hispanic, non-Hispanic white, non-Hispanic black, or others), marital status (married/living with partner, widowed/divorced/separated, or never married), educational level (high school and below, or some college and above) and PIR status (≤ 1.3, ≥ 1.3 to 3.5, or ≥ 3.5) were adjusted in model II. In model III, we further adjusted for ALT (continuous), AST (continuous), smoking status (never, current, or former), obesity (yes or no), T2DM (yes or no), hypertension (yes or no), IR (yes or no). The subgroup analyses were performed by the following covariates: age (< 40 years, ≥ 40 to 60 years, ≥ 60 years), obesity (yes, no), race/ethnicity, marital status, educational level and PIR status (≤ 1.3, ≥ 1.3 to 3.5, ≥ 3.5) and smoking status (never, current, former).

## Results

### Characteristics of participants in this study

The baseline characteristics of subjects excluded and included are summarized in Table 1. Of the 2149 participants, the mean (95% CI) age was 53.9 (95%CI 52.7–55.0), 686 (61.1%) were Non-Hispanic White. Of these, 884 (47.3%) participants were diagnosed NAFLD and 1042 (52.7%) have UI. Overall, the prevalence of UI was significantly higher among participants with NAFLD [490 (64.7%)] than among participants without NAFLD [552 (44.9%)]. Besides, the age was higher in participants with NAFLD (mean age, 57.2 years; 95% CI 55.6–58.8 years) than in those without (mean age, 51.7 years; 95%CI 50.4–53.0 years). Participants with NAFLD were more likely to have an educational level of some college and above [397 (41.9%) vs. 492 (34.5%)], to be Mexican American individuals [131 (10.1%) vs. 101 (5.1%)], to have diabetes [321 (32.4%) vs. 149 (7.7%)], to have hypertension [532 (57.6%) vs. 536 (35.1%)] and to have insulin resistance [342 (78.5%) vs. 220 (35.3%)]. Furthermore, compared with the participants with NAFLD, participants without NAFLD had significantly higher levels of BMI, waist circumference, ALT, AST, uric acid, triglycerides, total cholesterol, low-density lipoprotein-cholesterol (LDL-c) and had lower levels of high-density lipoprotein-cholesterol (HDL-c).

### Association between NAFLD and different types of UI

Multivariable logistic regression analysis was then constructed to investigate the relationship between NAFLD and different types of UI (as shown in Table 2). For individuals with NAFLD vs. those without NAFLD, the crude OR was 2.25 (95% CI, 1.59–3.18) for UI, 2.08 (95% CI, 1.43–3.01) for SUI, 1.81 (95% CI, 1.38–2.38) for UUI and 2.16 (95% CI, 1.53–3.07) for MUI. In the multivariable logistic adjusted models (model III) illustrated that patient with NAFLD had 93% and 55% higher odds of UI and UUI than those without NAFLD subjects [UI, OR: 1.93, 95%CI 1.23–3.02, *P* = 0.01; UUI, OR: 1.55, 95%CI 1.03–2.33, *P* = 0.03], while patients with NAFLD did not show an increased odds in SUI and MUI when compared with those without NAFLD subject (*P* > 0.05).

**Table 2:** Association of NAFLD with different types of UI among participants in the NHANES 2017-march 2020 (pre-pandemic) cycles

Types of UI	OR	95% CI	*P* value
UI			
Model I	2.25	1.59–3.18	0.00
Model II	2.15	1.46–3.17	0.00
Model III	1.93	1.23–3.02	0.01
SUI			
Model I	2.08	1.43–3.01	0.00
Model II	2.09	1.38–3.16	0.00
Model III	1.41	0.62–3.21	0.33
UUI			
Model I	1.81	1.38–2.38	0.00
Model II	1.63	1.16–2.28	0.01
Model III	1.55	1.03–2.33	0.03
MUI			
Model I	2.16	1.53–3.07	0.00
Model II	2.03	1.31–3.13	0.00
Model III	2.01	0.91–4.44	0.07

Abbreviations: OR: odds ratio; CI: confidence interval; UI: urinary incontinence; SUI: stress urinary incontinence; UUI: urge urinary incontinence

Model I: adjusted for none

Model II: adjusted for age, race/ethnicity, marital status, educational level and PIR status (≤ 1.3, ≥ 1.3 to 3.5, or ≥ 3.5)

Model III: adjusted for age, race/ethnicity, marital status, educational level and PIR status (≤ 1.3, ≥ 1.3 to 3.5, or ≥ 3.5), ALT, AST, smoking status (never, current, or former), obesity (yes or no), T2DM (yes or no), hypertension (yes or no), IR (yes or no)

**Table 3:** Stratified analyses of the associations between NAFLD and UI

		Participants^a^				
Subgroups		Without NAFLD	With NAFLD		Adjusted OR (95% CI)	*P* value
**Overall**		552/1265 (43.6%)	490/884 (55.4%)		1.93 (1.23–3.02)	0.01
**Age, years**						
< 40		90/357 (25.2%)	53/123 (43.1%)		1.16 (0.28–4.84)	0.81
≥ 40 to 60		153/398 (38.4%)	173/327 (52.9%)		1.99 (0.75–5.31)	0.13
≥ 60		309/510 (60.6%)	264/434 (60.8%)		2.20 (1.38–3.51)	0.01
**Obesity**						
No		337/888 (38.0%)	143/285 (50.2%)		2.69 (1.81-4.00)	0.01
Yes		208/369 (56.4%)	344/593 (58.0%)		1.43 (0.81–2.52)	0.28
**Race/ethnicity**						
Mexican American		41/101 (40.6%)	71/131 (54.2%)		1.55 (0.65–3.66)	0.43
Other Hispanic		54/143 (37.8%)	43/87 (49.4%)		2.25 (0.89–5.72)	0.23
Non-Hispanic White		228/542 (42.1%)	211/287 (73.5%)		2.27 (1.38–3.73)	0.08
Non-Hispanic Black		136/350 (38.9%)	106/225 (47.1%)		1.38 (0.36–5.35)	0.68
Other Race		93/272 (34.2%)	59/154 (38.3%)		1.38 (0.52–3.70)	0.58
**Educational level**						
High school and below		334/772 (43.3%)	292/484 (60.3%)		2.30 (1.56–3.38)	0.01
Some college and above		217/492 (44.1%)	198/397 (24.7%)		1.51 (0.75–3.05)	0.30
**Marital status**						
Married/ living with partner		270/670 (40.3%)	272/487 (55.9%)		2.06 (1.37–3.10)	0.03
Widowed/divorced/separated		216/376 (57.5%)	160/275 (58.2%)		1.32 (0.57–3.04)	0.55
Never married		63/216 (29.2%)	57/119 (47.9%)		4.31 (1.20–15.50)	0.09
**PIR**						
≤ 1.3		116/304 (38.2%)	122/236 (51.7%)		2.01 (0.70–5.72)	0.26
≥ 1.3 to 3.5		221/414 (53.4%)	190/315 (60.3%)		1.09 (0.58–2.07)	0.80
≥ 3.5		150/369 (40.7%)	131/226 (58.0%)		3.46 (1.82–6.58)	0.02
**Smoking status**						
Never		392/945 (41.5%)	331/639 (51.8%)		2.22 (1.44–3.42)	0.02
Current		54/118 (45.8%)	51/79 (64.6%)		1.86 (0.54–6.46)	0.38
Former		106/201 (52.7%)	108/166 (65.1%)		1.25 (0.62–2.53)	0.57

^a^ Data are presented as unweighted patients number/total number (unweighted percentage)

Each stratification was adjusted for age, race/ethnicity, marital status, educational level and PIR status (≤ 1.3, ≥ 1.3 to 3.5, or ≥ 3.5), ALT, AST, smoking status (never, current, or former), obesity (yes or no), T2DM (yes or no), hypertension (yes or no), IR (yes or no) except the stratification factor itself

### Subgroup analysis

To further investigate the association between NAFLD and UI, subgroup analyses were performed stratified by age, obesity, race/ethnicity, education level, marital status, PIR status and smoking status (As presented in Table 3). NAFLD was still significantly associated with UI, especially among those who without obesity (OR: 2.69, 95% CI 1.84-4.00), were older than 60 years (OR: 2.20, 95% CI 1.38–3.51), were non-Hispanic white (OR: 2.27, 95% CI 1.38–3.73), high school and below (OR: 2.30, 95% CI 1.56–3.05), married or living with partner (OR: 2.06, 95% CI 1.37–3.04) and never smoked participants (OR: 2.22, 95% CI 1.44–3.42).

## Discussion

The presented study investigated the relationship between NAFLD and different types of UI in a large U.S. population by analyzing the date of female participants over 20 years of age in 2017-March 2020 (pre-pandemic) NHANES cycles. We found that those who had been diagnosed with NAFLD had higher odds of UI and UUI than those without NAFLD. To further confirm the founding, age, race/ethnicity, marital status, educational level, PIR status, ALT, AST, smoking status, obesity, T2DM, hypertension and IR were adjusted in the multivariate logistic regression models. The results indicated that compared with those individuals without NAFLD, NAFLD patients were significantly associated with both UI (OR: 1.93, 95%CI 1.23–3.02, *P* = 0.01) and UUI (OR: 1.55, 95%CI 1.03–2.33, *P* = 0.03), but not with SUI and MUI.

There is no denying that excess weight and obesity are important risk factors for urinary incontinence in female [27, 28]. Besides, evidence in the literature supports the notion that MetS is a risk factor for UI [29, 30]. A cross-sectional study performed on 518 female with T2DM (aged 50–75 years) suggests that Mets specifically affects UI in diabetic female, probably by compounding the effect of peripheral neuropathy [31]. Its means that the association between Mets and UI is complex and not just affected by weight.

The findings of the presented study demonstrated that NAFLD is significantly correlated with UUI, and its potential mechanism needs to be further explored. We hypothesized that endothelial dysfunction, IR and the commodities might support this positive correlation. In a multivariable logistic regression model, some covariates including BMI were adjusted, and the results still illustrated that NAFLD was independently associated with the increased odds of UI and UUI. This suggested that the influence of NAFLD on UI, especially UUI, were independent of BMI itself. Considering that NAFLD patients were often characterized by IR, dyslipidemia, and glucose tolerance, these factors together constitute vascular injury and endothelial dysfunction [32, 33]. The changes of vascular mechanism might be involved in the pathophysiological process of UUI [34]. Furthermore, some comorbidities such as T2DM, pre-diabetes and dyslipidemia might also contribute to the pathophysiological process of both NAFLD and UUI [35, 36].

UUI is a subtype of UI, compared to SUI, UUI has a greater impact on patients’ physical and mental health. psychological distress and worsening quality of life [7, 37]. At the same time, the incidence of UUI is higher in female than in male, so further understanding of the pathogenic factors for female UUI may help to take effective preventive measures and formulate treatment strategies [38]. NAFLD is a clinical syndrome that coexists with obesity, hypertension, hyperglycemia (impaired glucose tolerance, impaired fasting glucose or diabetes), dyslipidemia and a combination of metabolic risk factors as one of the controllable and modifiable factors. Its pathogenesis mainly includes central obesity, IR, atherosclerosis and endothelial dysfunction [1, 16].

In subgroup analysis, NAFLD was still significantly associated with UI especially among those participants who were without obesity (OR: 2.69, 95% CI 1.84-4.00). Accumulating growing prospective cohort evidence that the increase of BMI is associated with various types of UI, as elevated BMI tends to increase intra-abdominal and intra-bladder pressure in patients with UI [27, 39, 40]. Therefore, combined with our results, we concluded that the influence on UI with or without NAFLD may not be significant for those patients with obesity. However, as for non-obese patients, the contribution of BMI to UI was relatively high. At this time, those lean-NAFLD patients may develop IR, vascular endothelial dysfunction and some complications, which together increase the incidence of UI [41].

Several limitations of the present study are worth mentioning. Firstly, due to the cross-sectional nature of this study, we cannot determine any causal association between NAFLD and UI. Further cohort studies may be warranted in the future. Secondly, the diagnose of NAFLD is based on VCTE, a non-invasive method, rather than liver biopsy. Nevertheless, VCTE is very suitable for large-scale population evaluation and show good test efficiency. Thirdly, although we have adjusted for many confounders and performed stratified analyses in the multivariable regression model, it is undeniable that there are still several potential confounders that are not considered. Fourthly, the diagnosis of UI is based on self-reported data, which may result in inaccurate results due to recall bias.

The results of the presented cross-sectional study indicate a positive association between NAFLD and UI in the U.S. female adults. Considering that NAFLD is a treatable and modifiable disease, active treatment of NAFLD may contribute to the treatment and management of UI.

**Table 1:** Base characteristics of participants with and without NAFLD in the NHANES 2017-march 2020 (pre-pandemic) cycles

Characteristic	Total (*n* = 2149)	Without NAFLD (*n* = 1265)	With NAFLD (*n* = 884)	*P* value
Age, years	53.9 (52.7–55.0)	51.7 (50.4–53.0)	57.2 (55.6–58.8)	< 0.01
Race/ethnicity				< 0.01
Mexican American	232 (7.1)	101 (5.1)	131 (10.1)	
Other Hispanic	230 (7.4)	143 (7.8)	87 (6.8)	
Non-Hispanic White	686 (61.1)	399 (61.7)	287 (60.3)	
Non-Hispanic Black	575 (12.4)	350 (13.0)	225 (11.4)	
Other Race	426 (12.0)	272 (12.5)	154 (11.4)	
Educational level				< 0.01
High school and below	1256 (62.6)	772 (65.5)	484 (58.1)	
Some college and above	889 (37.4)	492 (34.5)	397 (41.9)	
Marital status				0.24
Married/ living with partner	1157 (58.6)	670 (57.6)	487 (60.2)	
Widowed/divorced/separated	651 (27.3)	376 (26.9)	275 (27.9)	
Never married	335 (14.1)	216 (15.5)	119 (12.0)	
PIR				0.33
≤ 1.3	540 (20.0)	304 (19.0)	236 (21.5)	
≥ 1.3 to 3.5	729 (37.6)	414 (36.6)	315 (39.2)	
≥ 3.5	595 (42.4)	369 (44.4)	226 (39.4)	
BMI, kg/m^2^	29.8 (29.1–30.5)	26.8 (26.1–27.5)	34.5 (33.5–35.5)	< 0.01
Waist circumstance, cm	98.5 (96.8-100.2)	91.2 (89.4–92.9)	109.9 (107.9-111.9)	< 0.01
ALT, U/L	17.9 (17.5–18.4)	15.6 (15.1–16.1)	21.4 (20.2–22.5)	< 0.01
AST, U/L	19.3 (18.9–19.6)	18.5 (18.2–18.8)	20.4 (19.6–21.2)	< 0.01
Uric acid, µmol/L	285.9 (280.3-291.5)	267.1 (260.4-273.9)	314.3 (308.3-320.3)	< 0.01
Triglycerides, mmol/L	1.17 (1.12–1.22)	0.96 (0.91–1.01)	1.46 (1.36–1.56)	< 0.01
Total cholesterol, mmol/L	5.00 (4.92–5.08)	4.98 (4.89–5.07)	5.03 (4.92–5.13)	0.34
LDL cholesterol, mmol/L	2.92 (2.84-3.00)	2.91 (2.81-3.00)	2.94 (2.80–3.08)	0.66
HDL cholesterol, mmol/L	1.50 (1.47–1.53)	1.59 (1.56–1.62)	1.37 (1.34–1.41)	< 0.01
Smoking status				0.66
Never	1584 (71.0)	945 (72.0)	639 (69.6)	
Current	197 (9.8)	118 (9.8)	79 (9.8)	
Former	367 (19.2)	201 (18.3)	166 (20.6)	
Diabetes	470 (17.4)	149 (7.7)	321 (32.4)	< 0.01
Hypertension	1068 (44.0)	536 (35.1)	532 (57.6)	< 0.01
Insulin resistance	562 (53.0)	220 (35.3)	342 (78.5)	< 0.01
UI	1042 (52.7)	552 (44.9)	490 (64.7)	< 0.01
SUI	920 (45.7)	459 (38.6)	461 (56.6)	< 0.01
UUI	754 (31.7)	386 (26.6)	368 (39.6)	< 0.01
MUI	429 (58.3)	192 (14.3)	237 (26.5)	< 0.01

Characteristics of participants are described as means (95% CIs) for continuous variables and unweighted numbers (weighted percentages) for categorical variables

Abbreviation: PIR: family poverty income ratio; BMI, body mass index; ALT, alanine aminotransferase; AST, aspartate aminotransferase. LDL, low-density lipoprotein; HDL, high-density lipoprotein

## Data Availability

Publicly available datasets were analyzed in this study. This data can be found here: The National Health and Nutrition Examination Survey dataset at https://www.cdc.gov/nchs/nhanes/index.htm.

## References

[1] Chalasani N, Younossi Z, Lavine JE, Diehl AM, Brunt EM, Cusi K, Charlton M, Sanyal AJ (2012). The diagnosis and management of non-alcoholic fatty liver disease: practice guideline by the American Gastroenterological Association, American Association for the Study of Liver Diseases, and American College of Gastroenterology. Gastroenterology.

[2] Riazi K, Azhari H, Charette JH, Underwood FE, King JA, Afshar EE, Swain MG, Congly SE, Kaplan GG, Shaheen AA (2022). The prevalence and incidence of NAFLD worldwide: a systematic review and meta-analysis. Lancet Gastroenterol Hepatol.

[3] Ciardullo S, Carbone M, Invernizzi P, Perseghin G (2022). Impact of the new definition of metabolic dysfunction-associated fatty liver disease on detection of significant liver fibrosis in US adolescents. Hepatol Commun.

[4] Younossi ZM, Blissett D, Blissett R, Henry L, Stepanova M, Younossi Y, Racila A, Hunt S, Beckerman R (2016). The economic and clinical burden of nonalcoholic fatty liver disease in the United States and Europe. Hepatology.

[5] Wong VW, Wong GL, Chan RS, Shu SS, Cheung BH, Li LS, Chim AM, Chan CK, Leung JK, Chu WC (2018). Beneficial effects of lifestyle intervention in non-obese patients with non-alcoholic fatty liver disease. J Hepatol.

[6] Wei X, Lin B, Huang Y, Yang S, Huang C, Shi L, Liu D, Zhang P, Lin J, Xu B (2023). Effects of Time-restricted eating on nonalcoholic fatty liver disease: the TREATY-FLD randomized clinical trial. JAMA Netw Open.

[7] Gacci M, Sakalis VI, Karavitakis M, Cornu JN, Gratzke C, Herrmann TRW, Kyriazis I, Malde S, Mamoulakis C, Rieken M (2022). European Association of Urology Guidelines on male urinary incontinence. Eur Urol.

[8] Lukacz ES, Santiago-Lastra Y, Albo ME, Brubaker L (2017). Urinary incontinence in women: a review. JAMA.

[9] Cao C, Zhang C, Sriskandarajah C, Xu T, Gotto G, Sutcliffe S, Yang L (2022). Trends and racial disparities in the prevalence of urinary incontinence among men in the USA, 2001–2020. Eur Urol Focus.

[10] Abufaraj M, Xu T, Cao C, Siyam A, Isleem U, Massad A, Soria F, Shariat SF, Sutcliffe S, Yang L (2021). Prevalence and trends in urinary incontinence among women in the United States, 2005–2018. Am J Obstet Gynecol.

[11] Li D, Xu Y, Nie Q, Li Y, Mao G (2017). Predictors of urinary incontinence between abdominal obesity and non-obese male adults. Postgrad Med.

[12] Foley AL, Loharuka S, Barrett JA, Mathews R, Williams K, McGrother CW, Roe BH (2012). Association between the Geriatric Giants of urinary incontinence and falls in older people using data from the Leicestershire MRC Incontinence Study. Age Ageing.

[13] Buckley BS, Lapitan MC (2010). Prevalence of urinary incontinence in men, women, and children–current evidence: findings of the Fourth International Consultation on Incontinence. Urology.

[14] Weiß J, Rau M, Geier A (2014). Non-alcoholic fatty liver disease: epidemiology, clinical course, investigation, and treatment. Dtsch Arztebl Int.

[15] Younossi Z, Anstee QM, Marietti M, Hardy T, Henry L, Eslam M, George J, Bugianesi E (2018). Global burden of NAFLD and NASH: trends, predictions, risk factors and prevention. Nat Rev Gastroenterol Hepatol.

[16] Francque SM, Marchesini G, Kautz A, Walmsley M, Dorner R, Lazarus JV, Zelber-Sagi S, Hallsworth K, Busetto L, Frühbeck G (2021). Non-alcoholic fatty liver disease: a patient guideline. JHEP Rep.

[17] EASL-EASD-EASO (2016). Clinical practice guidelines for the management of non-alcoholic fatty liver disease. J Hepatol.

[18] Castera L, Friedrich-Rust M, Loomba R (2019). Noninvasive Assessment of Liver Disease in patients with nonalcoholic fatty liver disease. Gastroenterology.

[19] Barr RG, Ferraioli G, Palmeri ML, Goodman ZD, Garcia-Tsao G, Rubin J, Garra B, Myers RP, Wilson SR, Rubens D et al. Elastography Assessment of Liver Fibrosis: Society of Radiologists in Ultrasound Consensus Conference Statement. *Radiology* 2015, 276(3):845–861. 10.1148/radiol.2015150619 26079489

[20] Eddowes PJ, Sasso M, Allison M, Tsochatzis E, Anstee QM, Sheridan D, Guha IN, Cobbold JF, Deeks JJ, Paradis V (2019). Accuracy of FibroScan Controlled Attenuation parameter and liver stiffness measurement in assessing steatosis and fibrosis in patients with nonalcoholic fatty liver disease. Gastroenterology.

[21] Ciardullo S, Oltolini A, Cannistraci R, Muraca E, Perseghin G (2022). Sex-related association of nonalcoholic fatty liver disease and liver fibrosis with body fat distribution in the general US population. Am J Clin Nutr.

[22] Wu S, Wu F (2023). Association of urinary incontinence with depression among men: a cross-sectional study. BMC Public Health.

[23] Ogden CL, Carroll MD, Fakhouri TH, Hales CM, Fryar CD, Li X, Freedman DS (2018). Prevalence of obesity among youths by Household Income and Education Level of Head of Household - United States 2011–2014. MMWR Morb Mortal Wkly Rep.

[24] 2 (2020). Classification and diagnosis of diabetes: standards of Medical Care in Diabetes-2020. Diabetes Care.

[25] Muntner P, Hardy ST, Fine LJ, Jaeger BC, Wozniak G, Levitan EB, Colantonio LD (2020). Trends in blood pressure control among US adults with hypertension, 1999–2000 to 2017–2018. JAMA.

[26] Matthews DR, Hosker JP, Rudenski AS, Naylor BA, Treacher DF, Turner RC (1985). Homeostasis model assessment: insulin resistance and beta-cell function from fasting plasma glucose and insulin concentrations in man. Diabetologia.

[27] Khullar V, Sexton CC, Thompson CL, Milsom I, Bitoun CE, Coyne KS (2014). The relationship between BMI and urinary incontinence subgroups: results from EpiLUTS. Neurourol Urodyn.

[28] Lamerton, Mielke, Brown (2021). Urinary incontinence, body mass index, and physical activity in young women. Am J Obstet Gynecol.

[29] Ströher RLM, Sartori MGF, Takano CC, de Araújo MP, Girão M (2020). Metabolic syndrome in women with and without stress urinary incontinence. Int Urogynecol J.

[30] Otunctemur A, Dursun M, Ozbek E, Sahin S, Besiroglu H, Koklu I, Erkoc M, Danis E, Bozkurt M (2014). Impact of metabolic syndrome on stress urinary incontinence in pre- and postmenopausal women. Int Urol Nephrol.

[31] Tai HC, Chung SD, Ho CH, Tai TY, Yang WS, Tseng CH, Wu HP, Yu HJ (2010). Metabolic syndrome components worsen lower urinary tract symptoms in women with type 2 diabetes. J Clin Endocrinol Metab.

[32] Nasiri-Ansari N, Androutsakos T, Flessa CM, Kyrou I, Siasos G, Randeva HS, Kassi E, Papavassiliou AG. Endothelial Cell Dysfunction and Nonalcoholic Fatty Liver Disease (NAFLD): A Concise Review. *Cells* 2022, 11(16). 10.3390/cells11162511 PMC9407007 36010588

[33] Stahl EP, Dhindsa DS, Lee SK, Sandesara PB, Chalasani NP, Sperling LS (2019). Nonalcoholic fatty liver Disease and the heart: JACC state-of-the-art review. J Am Coll Cardiol.

[34] Tsui A, Kuh D, Cardozo L, Davis D (2018). Vascular risk factors for male and female urgency urinary incontinence at age 68 years from a British birth cohort study. BJU Int.

[35] Yu HJ, Liu CY, Lee KL, Lee WC, Chen TH (2006). Overactive bladder syndrome among community-dwelling adults in Taiwan: prevalence, correlates, perception, and treatment seeking. Urol Int.

[36] Brown JS, Vittinghoff E, Lin F, Nyberg LM, Kusek JW, Kanaya AM (2006). Prevalence and risk factors for urinary incontinence in women with type 2 diabetes and impaired fasting glucose: findings from the National Health and Nutrition Examination Survey (NHANES) 2001–2002. Diabetes Care.

[37] Azuma R, Murakami K, Iwamoto M, Tanaka M, Saita N, Abe Y (2008). Prevalence and risk factors of urinary incontinence and its influence on the quality of life of Japanese women. Nurs Health Sci.

[38] Agarwal A, Eryuzlu LN, Cartwright R, Thorlund K, Tammela TL, Guyatt GH, Auvinen A, Tikkinen KA (2014). What is the most bothersome lower urinary tract symptom? Individual- and population-level perspectives for both men and women. Eur Urol.

[39] Kuh D, Cardozo L, Hardy R (1999). Urinary incontinence in middle aged women: childhood enuresis and other lifetime risk factors in a British prospective cohort. J Epidemiol Community Health.

[40] Noblett KL, Jensen JK, Ostergard DR (1997). The relationship of body mass index to intra-abdominal pressure as measured by multichannel cystometry. Int Urogynecol J Pelvic Floor Dysfunct.

[41] Fuchs A, Samovski D, Smith GI, Cifarelli V, Farabi SS, Yoshino J, Pietka T, Chang SW, Ghosh S, Myckatyn TM (2021). Associations among adipose tissue immunology, inflammation, exosomes and insulin sensitivity in people with obesity and nonalcoholic fatty liver disease. Gastroenterology.

